# Depth-dependent ratcheting strains of young and adult articular cartilages by experiments and predictions

**DOI:** 10.1186/s12938-019-0705-7

**Published:** 2019-07-30

**Authors:** Li-Lan Gao, Xiang-Long Lin, Dong-Dong Liu, Ling Chen, Chun-Qiu Zhang, Hong Gao

**Affiliations:** 1 Tianjin Key Laboratory for Advanced Mechatronic System Design and Intelligent Control, School of Mechanical Engineering, Tianjin University of Technology, Tianjin, China; 2 National Demonstration Center for Experimental Mechanical and Electrical Engineering Education (Tianjin University of Technology), Tianjin, China; 3 School of Chemical Engineering and Technology, Tianjin University, Tianjin, China

**Keywords:** Young and adult articular cartilages, Ratcheting strain, Loading conditions, Hysteresis response, Depth dependent, Ratcheting model

## Abstract

**Background:**

Ratcheting strain is produced due to the repeated accumulation of compressive strain in cartilage and may be a precursor to osteoarthritis. The aim of this study was to investigate the ratcheting behaviors of young and adult articular cartilages under cyclic compression by experiments and theoretical predictions.

**Methods:**

A series of uniaxial cyclic compression tests were conducted for young and adult cartilage, and the effects of different loading conditions on their ratcheting behaviors were probed. A theoretical ratcheting model was constructed and applied to predict the ratcheting strains of young and adult cartilages with different loading conditions.

**Results:**

Ratcheting strains of young and adult cartilages rapidly increased at the initial stage, followed by a slower increase in subsequent stages. The strain accumulation value and its rate for young cartilage were greater than them for adult cartilage. The ratcheting strains of the two groups of cartilage samples decreased with increasing stress rate, while they increased with increasing stress amplitude. As the stress amplitude increased, the gap between the ratcheting strains of young and adult cartilages increased gradually. The ratcheting strains of young and adult cartilages decreased along the cartilage depth from the surface to the deep layer. The ratcheting strains of different layers increased with the compressive cycle, and the difference among the three layers was noticeable. Additionally, the theoretical predictions agreed with the experimental data.

**Conclusions:**

Overall, the ratcheting behavior of articular cartilage is affected by the degree of articular cartilage maturation.

## Background

Ratcheting behavior is an inelastic cyclic deformation characteristic of material under asymmetric stress cycling. It is an important factor that must be considered in structural safety and life assessment. The physiological loads of cartilage are complex; however, cyclic compression is the main type of loading mechanism for articular cartilage during daily activities, such as walking and running. During cyclic compression, articular cartilage undergoes an asymmetric stress-control cycle load with an average stress greater than zero, and thus, ratcheting deformation of articular cartilage is produced. In a single cycle, the resulting strain is not large, but this small strain gradually increases with increasing cycle number. When the strain accumulates to a certain extent, fatigue damage of cartilage can be caused, and osteoarthritis can occur in the joint. Therefore, it is worthwhile to study the ratcheting behavior of articular cartilage under cyclic loading.

Currently, there is much research on the mechanical properties of cartilage under cyclic loading. Song et al. investigated the dynamic deformation behavior and cumulative strain of tibial articular cartilage in knee joints under cyclic loads after meniscectomy [1]. Speirs et al. investigated the stress and pressure distributions in the solid and fluid phases of cartilage under cyclic load by applying the finite element method [2]. Sadeghi et al. found that the loading frequencies associated with normal, above normal and traumatic frequencies affected the surface damage of bovine articular cartilage [3]. Suh mathematically simulated the dynamic behavior of a cylindrical disk subjected to cyclic compression and analyzed the frequency-dependent characteristics of dilatation, hydrostatic pressure and interstitial fluid velocity [4]. Van Turnhout et al. found that the collagen fibril network is an important factor for the depth-dependent mechanical behavior of adult articular cartilage [5]. Vikingsson et al. studied the long-term mechanical behaviors of porous scaffolds for the regeneration of articular cartilage under cyclic compression [6]. Gao et al. investigated ratcheting behavior for online soaked and unsoaked cartilage samples under cyclic compression [7]. Overall, these studies have focused on the mechanical properties of adult cartilage under cyclic loading. However, there is little research on the mechanical behavior of young cartilage under cyclic loading.

As we know, the composition and structure of articular cartilage change at the range of its development and growth, which can cause the alteration of mechanical properties in cartilage. Articular cartilage consists of a number of chondrocytes embedded in a porous extracellular matrix (ECM). Collagen is the most abundant ECM component (≈ 75% of dry weight). Both the predominant orientation in the collagen network and the amount of collagen in the network affect the mechanical behaviors of cartilage, and thus the functioning of articular cartilage [5, 8–10]. Van Turnhout et al. found that collagen content increases with age between birth and maturity [11], which can cause the alteration of mechanical properties in cartilage. The tensile moduli and strength of cartilage samples increased by an average of 391–1060% with increase of the collagen concentration when growing from the fetus to the adult [12]. Articular cartilage was found to become stiffer and less permeable with age, whilst collagen content significantly increased [13]. Hence, it is necessary to investigate the mechanical properties of cartilage at different ages under cyclic loading and to probe the biomechanical relationship between young and adult cartilage.

Since ratcheting strain can cause the fatigue damage of cartilage and lead to osteoarthritis in the joint, we investigated the ratcheting behaviors of young and adult cartilages under cyclic compression. The evolution trends of their stress–strain hysteresis loop and ratcheting strain were obtained with different stress rates and stress amplitudes. We probed the depth-dependent ratcheting behaviors of young and adult cartilages. In addition, the ratcheting model was constructed and applied to predict their ratcheting strains with different loading conditions. These findings can provide a reference for preventing the cartilage’s fatigue damage.

## Materials and methods

**Fig. 1:**
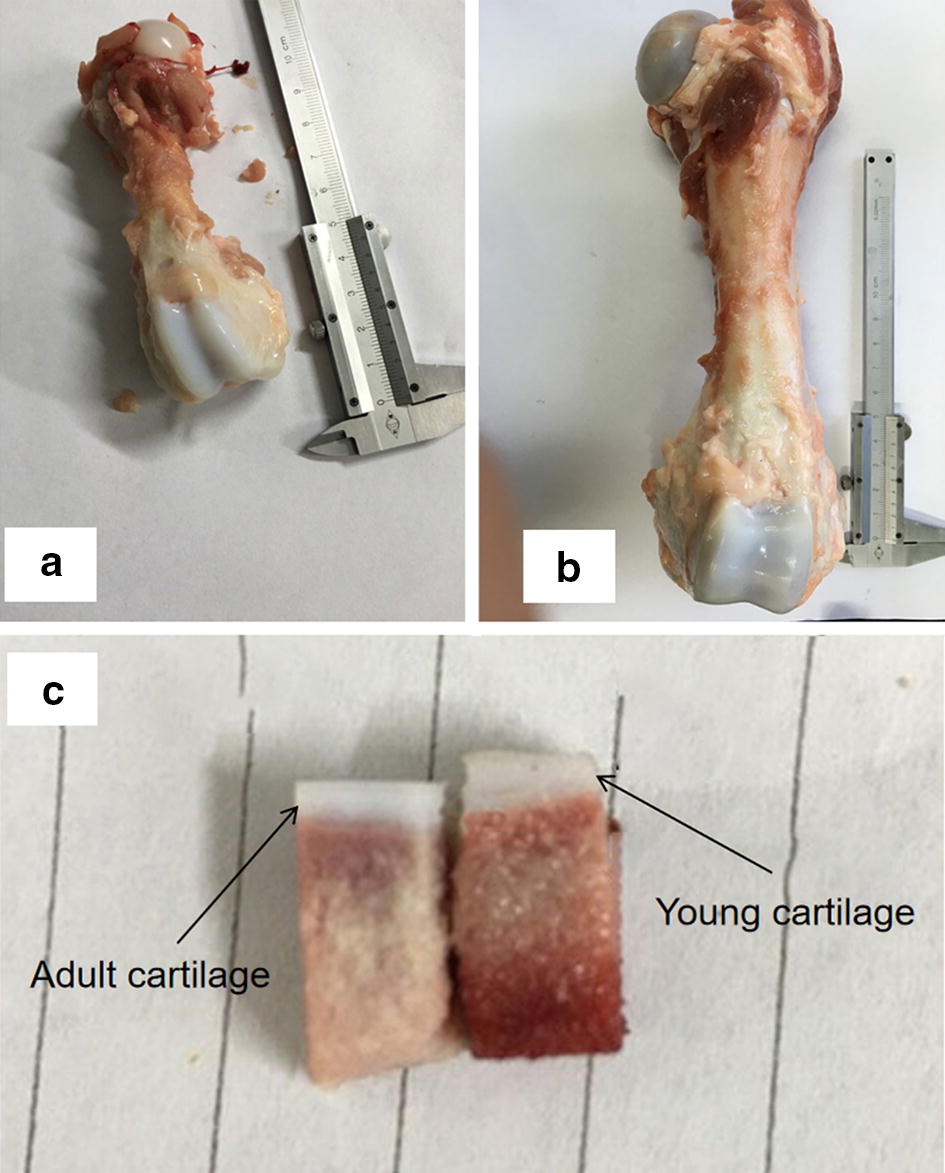
Fresh material samples of (**a**) femur of young pig; (**b**) femur of adult pig; (**c**) young and adult articular cartilages

In this study, the experimental material was the fresh articular cartilage which was obtained from the femur of 1-month-old pig (Young pig) and 6-month-old pig (Adult pig) as shown in Fig. 1a, b. The steel trephine was used to harvest the full-thickness cartilage samples with subchondral bone. For compression to be distributed on the whole cartilage surface, the cutting core axis was perpendicular to the articular surface. Each cartilage sample was made into a cuboid with square base of 5 mm length. The thickness of young cartilage was 2.5–3.5 mm and the thickness of adult cartilage was 1.8–2.2 mm as shown in Fig. 1c. The thickness of subchondral bone was about 15 mm (used for holding). After preparation, the samples were wrapped in sterile gauze and dipped into physiological saline so as to maintain the physiological environment of cartilage. Approval for the animal experiments was obtained from the Institutional Animal Care and Use Committee at Tianjin.

**Fig. 2:**
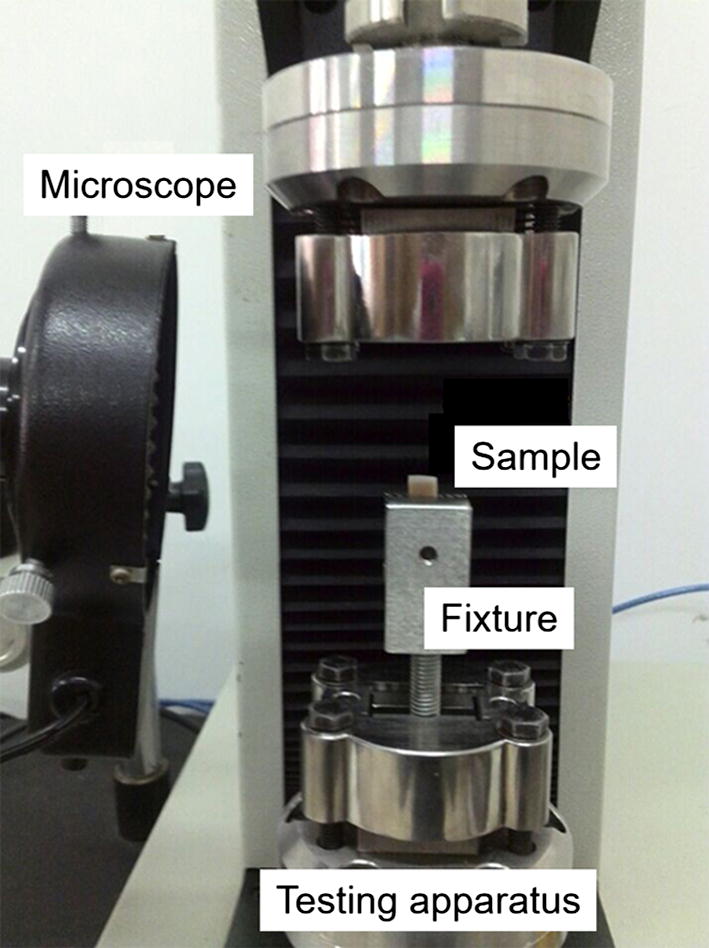
The experimental equipment

**Fig. 3:**
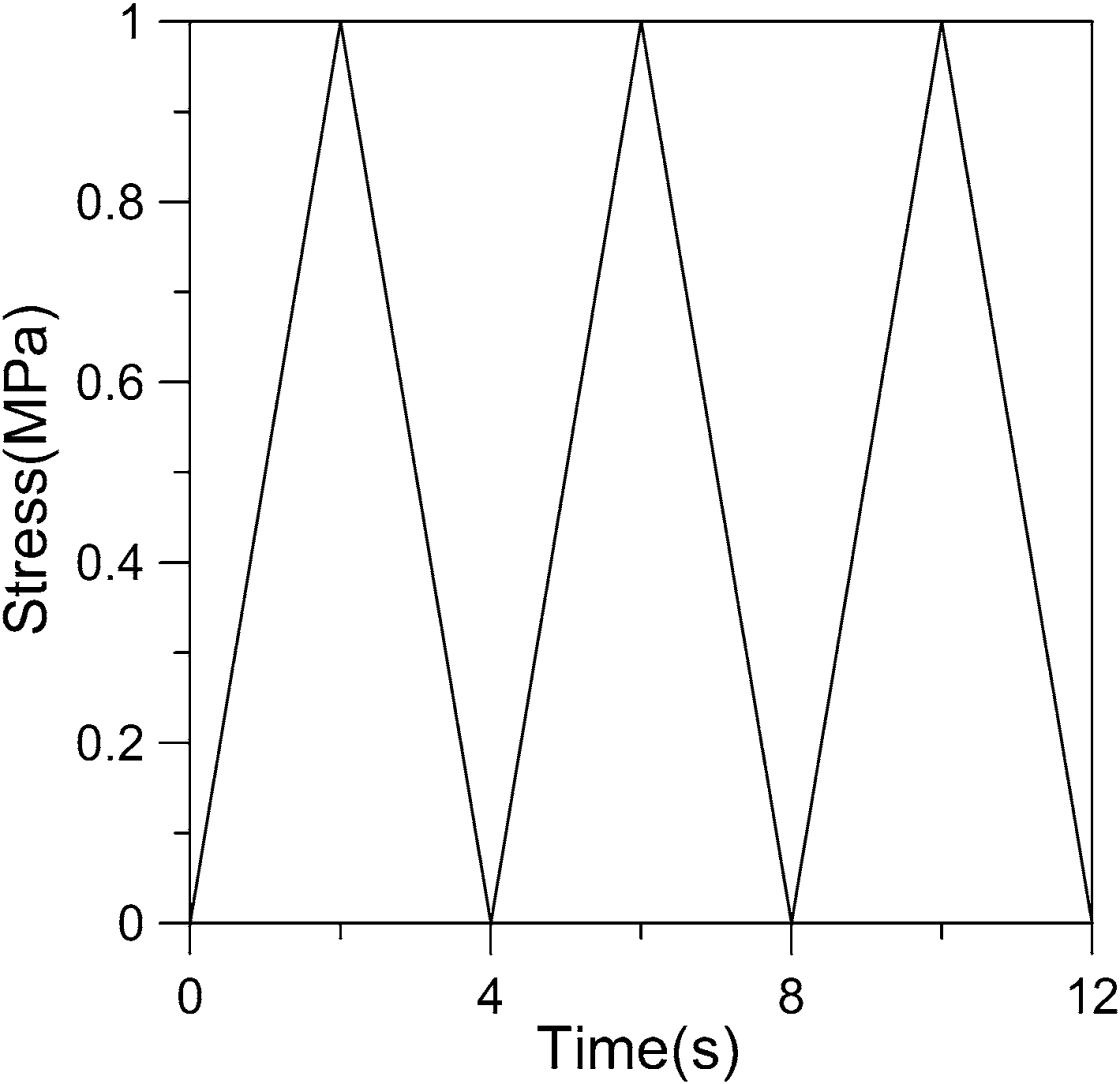
Triangular wave loading curve

The uniaxial ratcheting tests of young and adult articular cartilages were performed, respectively, under cyclic loading with typical triangular wave load. Figure 2 shows the experimental equipment and Fig. 3 shows the loading curve. The effects of stress amplitude and stress rate on ratcheting strain were probed for the young and adult cartilage samples and the detailed loading conditions are shown in Table 1. To avoid the random error, three independent samples were conducted for each group.

**Table 1:** Loading conditions of ratcheting test for young and adult cartilages

Specimen ID	Stress amplitude (MPa)	Stress rate (MPa/s)	Sample
SP1–SP3	1	0.5	Adult cartilage
SP4–SP6	1	0.5	Youth cartilage
SP7–SP9	1	1	Adult cartilage
SP10–SP12	1	1	Youth cartilage
SP13–SP15	1	2	Adult cartilage
SP16–SP18	1	2	Youth cartilage
SP19–SP21	0.5	0.5	Adult cartilage
SP22–SP24	0.5	0.5	Youth cartilage
SP25–SP27	1	0.5	Adult cartilage
SP28–SP30	1	0.5	Youth cartilage
SP31–SP33	2	0.5	Adult cartilage
SP34–SP36	2	0.5	Youth cartilage

The optimized digital image correlation (DIC) technique was applied to study the ratcheting behaviors of different layers of the young and adult articular cartilages. Here the iron oxide nanoparticles with a diameter of 100 nm were used as markers on cartilage samples. The images were first captured for young and adult cartilage samples prior to cyclic loading. The structure of adult cartilage can be divided into three distinct layers: superficial layer of about 15%, middle layer of about 55% and deep zone of about 30% to the full thickness of cartilage by analyzing images. Referring to the layer structure of adult cartilage, the young cartilage was also divided into three layers and the thickness ratio of each layer was the same as that of adult group. The images of samples were simultaneously captured per 2 s during the whole cyclic loading process. By applying the analysis software of DIC technique, the change of markers on the captured digitized images of cartilage sample was tracked, and then we can obtain the corresponding displacements or strain of different layers of young and adult cartilage samples.

### Description of ratcheting model for articular cartilage

The uniaxial ratcheting strain of material can be expressed as follows if the effects of some factors, such as temperature, creep and loading history, on ratcheting strain are not taken into account [14]:

1 $$\bar{\varepsilon }_{\text{r}} = 1 - \alpha N^{\beta } ,$$

where $\bar{\varepsilon }_{\text{r}} = \varepsilon_{\text{r}} /\varepsilon_{\text{r}}^{\text{s}}$, $\varepsilon_{\text{r}}$ is the ratcheting strain and $\varepsilon_{\text{r}}^{\text{s}}$ is the saturated ratcheting strain. $N$ is the number of cycles. $\alpha$ and $\beta$ are the material constants.

Based on a series of uniaxial ratcheting tests [15], it is found that there was a certain relationship between the saturated ratcheting strain and ratcheting stress of material, and proposed the saturated ratcheting strain model to describe their relationship. The following equation shows the saturated ratcheting strain model:

2 $$\varepsilon_{\text{r}}^{\text{s}} = a\left( {\sigma_{\text{r}} - \sigma^{\prime}_{\text{r}} } \right)^{2} + b\left( {\sigma_{\text{r}} - \sigma^{\prime}_{\text{r}} } \right),$$

where $\sigma_{\text{r}}$ is the ratcheting stress which refers to peak stress in a cycle. $\sigma^{\prime}_{\text{r}}$ is the maximum stress without ratcheting strain, which is defined as the ratcheting stress threshold. $a$ and $b$ are the parameters, being related to stress rate.

By substituting Eq. (2) into Eq. (1), the universal ratcheting model is shown as follows:

3 $$\varepsilon_{\text{r}} = \left[ {a\left( {\sigma_{\text{r}} - \sigma^{\prime}_{\text{r}} } \right)^{2} + b\left( {\sigma_{\text{r}} - \sigma^{\prime}_{\text{r}} } \right)} \right]\left( {1 - \alpha N^{\beta } } \right).$$

The ratcheting strain of articular cartilage is influenced by loading conditions such as peak stress, stress amplitude or stress rate. The effect of peak stress or stress amplitude on ratcheting strain of cartilage has been considered as shown in ratcheting model (Eq. (3)). However, this model cannot predict the ratcheting behavior of cartilage with different stress rates. So we modified the model by associating parameters $a$ and $b$ with stress rate. Using the least square method, the relationships between them are acquired, respectively,

4 $$a = \mu (\ln \dot{\sigma }) + \gamma ,$$

5 $$b = \kappa \dot{\sigma }^{\omega } ,$$

where $\dot{\sigma }$ is the stress rate. $\mu$, $\gamma$, $\kappa$ and $\omega$ are the material constants. Thus, the ratcheting strain of young and adult cartilages with different stress amplitudes and stress rates can be predicted based on Eqs. (3)–(5).

To predict the ratcheting strain of different layers of young and adult cartilage, the above ratcheting model was modified by adding the function $f(Z)$ related to the normalized depth of cartilage, and the modified model is shown in the following equation:

6 $$\varepsilon_{r} = \left[ {a\left( {\sigma_{r} - \sigma^{\prime}_{r} } \right)^{2} + b\left( {\sigma_{r} - \sigma^{\prime}_{r} } \right)} \right]\left( {1 - \alpha N^{\beta } } \right)f(Z),$$

where $Z$ is the normalized depth of cartilage, and the relationships between $f(Z)$ and normalized depth are fitted:

7 $${\text{Young cartilage:}}\,f(Z) = - 1.040Z + 1.075;$$

8 $${\text{Adult cartilage:}}\,f(Z) = - 0.983Z + 1.196.$$

## Results

### Ratcheting behaviors of young and adult articular cartilages

**Fig. 4:**
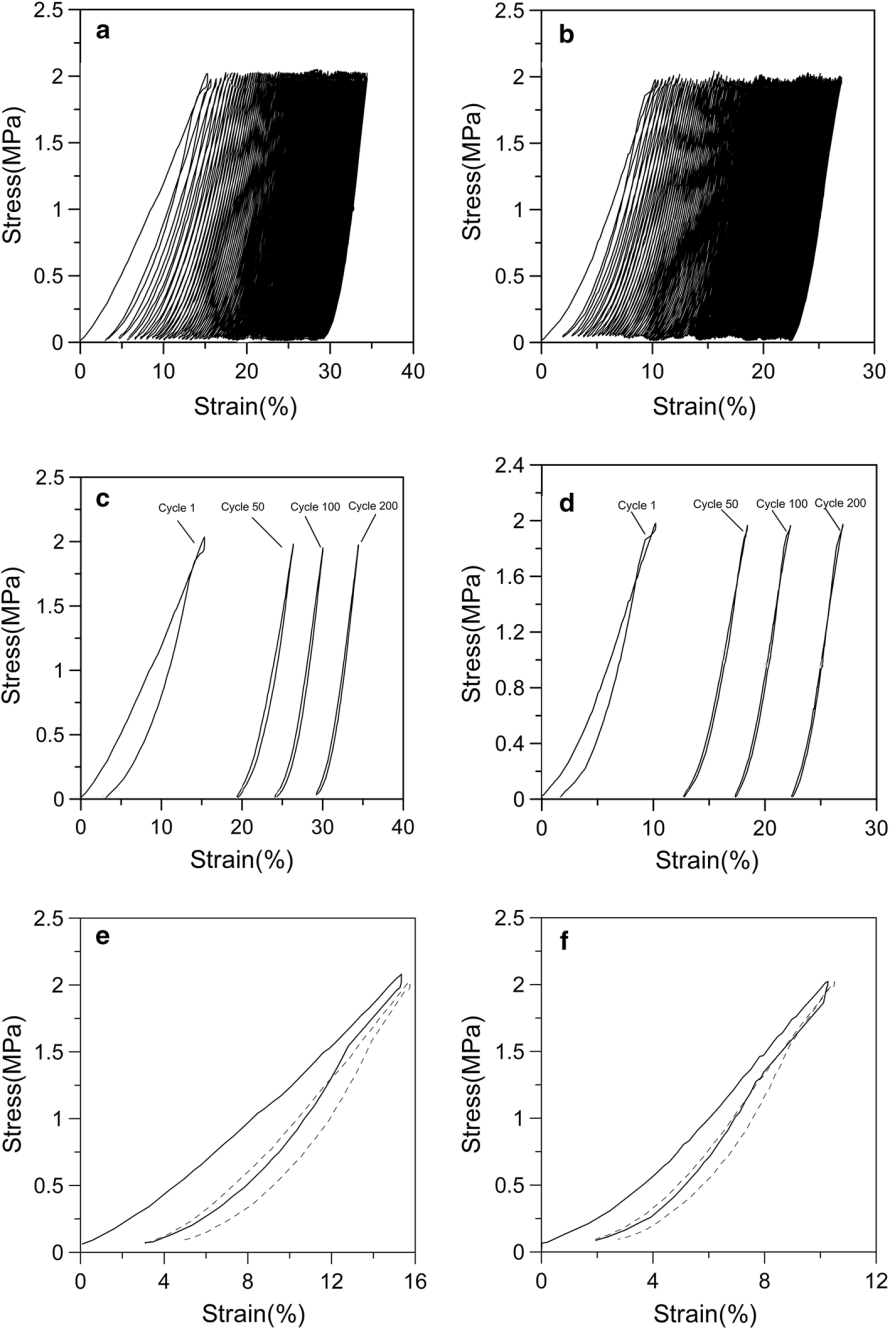
Stress–strain hysteresis loop with stress amplitude of 1 MPa and stress rate of 2 MPa/s. **a** Young cartilage; **b** adult cartilage; **c** young cartilage under different cycles; **d** adult cartilage under different cycles; **e** young cartilage under the first two cycles; **f** adult cartilage under the first two cycles

The stress–strain curves of young and adult articular cartilages were obtained under cyclic compression and Fig. 4a, b shows the stress–strain curves for young and adult cartilages with stress amplitude of 1 MPa and stress rate of 2 MPa/s. It can be seen that the hysteresis loops within 200 cycles are not closed, which will result in the ratcheting response of the material. To observe the hysteresis loop clearly, the stress–strain curves with 1 cycle, 50 cycles, 100 cycles and 200 cycles are given in Fig. 4c, d. The stress–strain responses of young and adult cartilage samples become denser with the superposition of cycle, which indicates that the stacking rate of deformation is gradually decreasing. It is noted that the hysteresis loop area of young cartilage in the first 50 laps is significantly larger than that of adult cartilage. Figure 4e, f shows that in the first two loading cycles, the unloading part of the first cycle stress–strain hysteresis curve corresponds to the loading part of the second cycle, and the tendency of the loading and unloading curves to approach gradually is clearly seen.

Under the cyclic stress, articular cartilage produces multiple cumulative progressive deformations. In this study, this deformation of articular cartilage was defined by ratcheting strain, and the expression of ratcheting strain is shown as follows:

9 $$\varepsilon_{\text{r}} = \frac{{\varepsilon_{\text{max} } + \varepsilon_{\text{min} } }}{2},$$

where $\varepsilon_{\text{r}}$ is the ratcheting strain, $\varepsilon_{ \text{max} }$ and $\varepsilon_{ \text{min} }$ are the maximum strain value and the minimum strain value during cyclic loading, respectively.

**Fig. 5:**
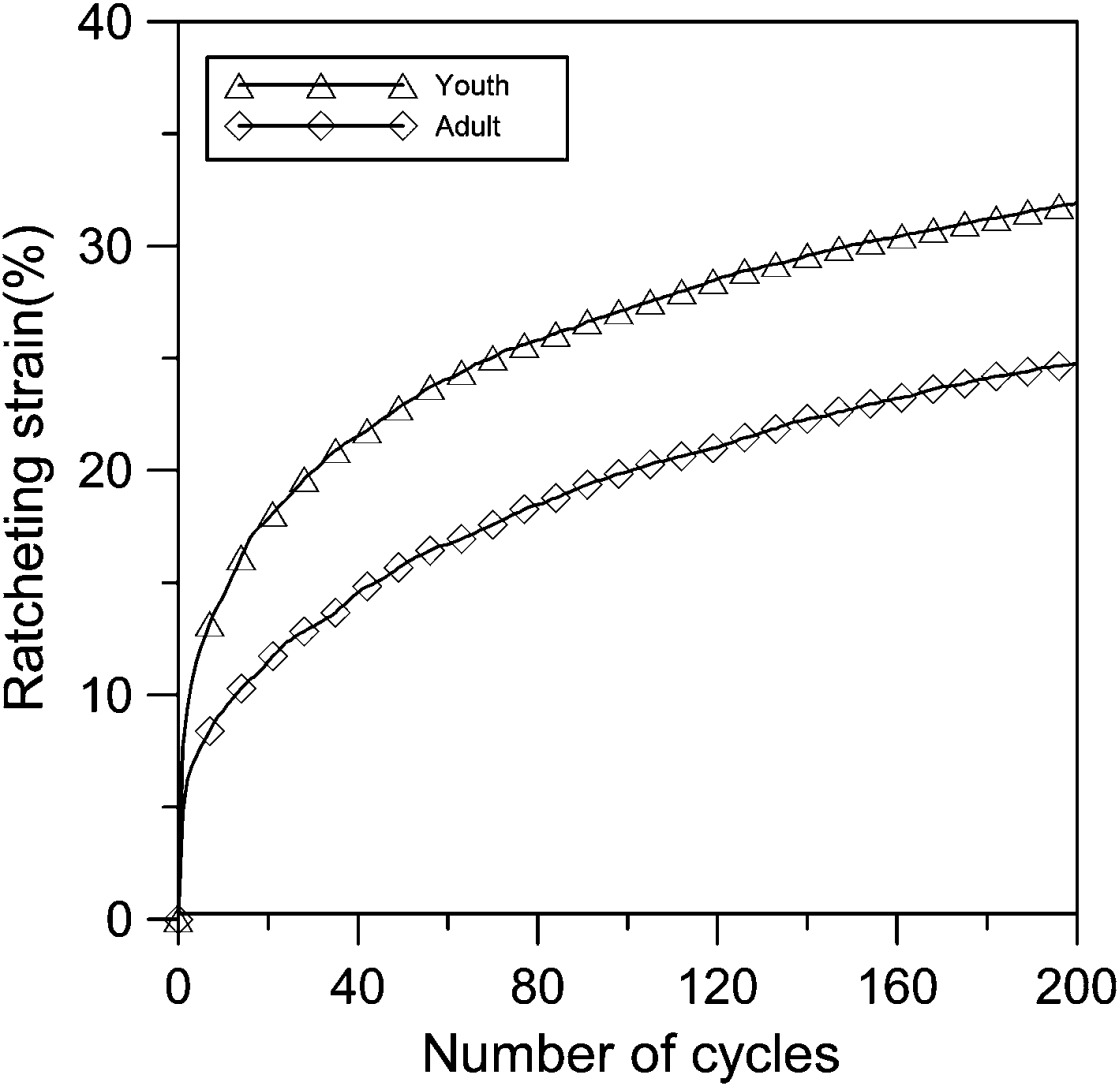
The ratcheting strains of young and adult cartilages with the stress amplitude of 1 MPa and stress rate of 2 MPa/s

Figure 5 shows the ratcheting strains of young and adult cartilages with cyclic compression going on when applying the stress amplitude of 1 MPa and stress rate of 2 MPa/s. Because the number of cycles is only 200, the ratcheting behaviors of both young and adult articular cartilages show the two stages. The first stage is very short, and the ratcheting strains increase rapidly. The second stage is relatively long and the ratcheting strains grow slowly, showing a smaller strain accumulation rate. It is also noted that the ratcheting strain of young cartilage in the same number of cycles is larger than that of adult cartilage. The reason is that adult articular cartilage has less liquid content and more collagen content so that cartilage’s resistance to deformation is relatively strong.

### Effect of stress rate on ratcheting strain of young and adult articular cartilages

**Fig. 6:**
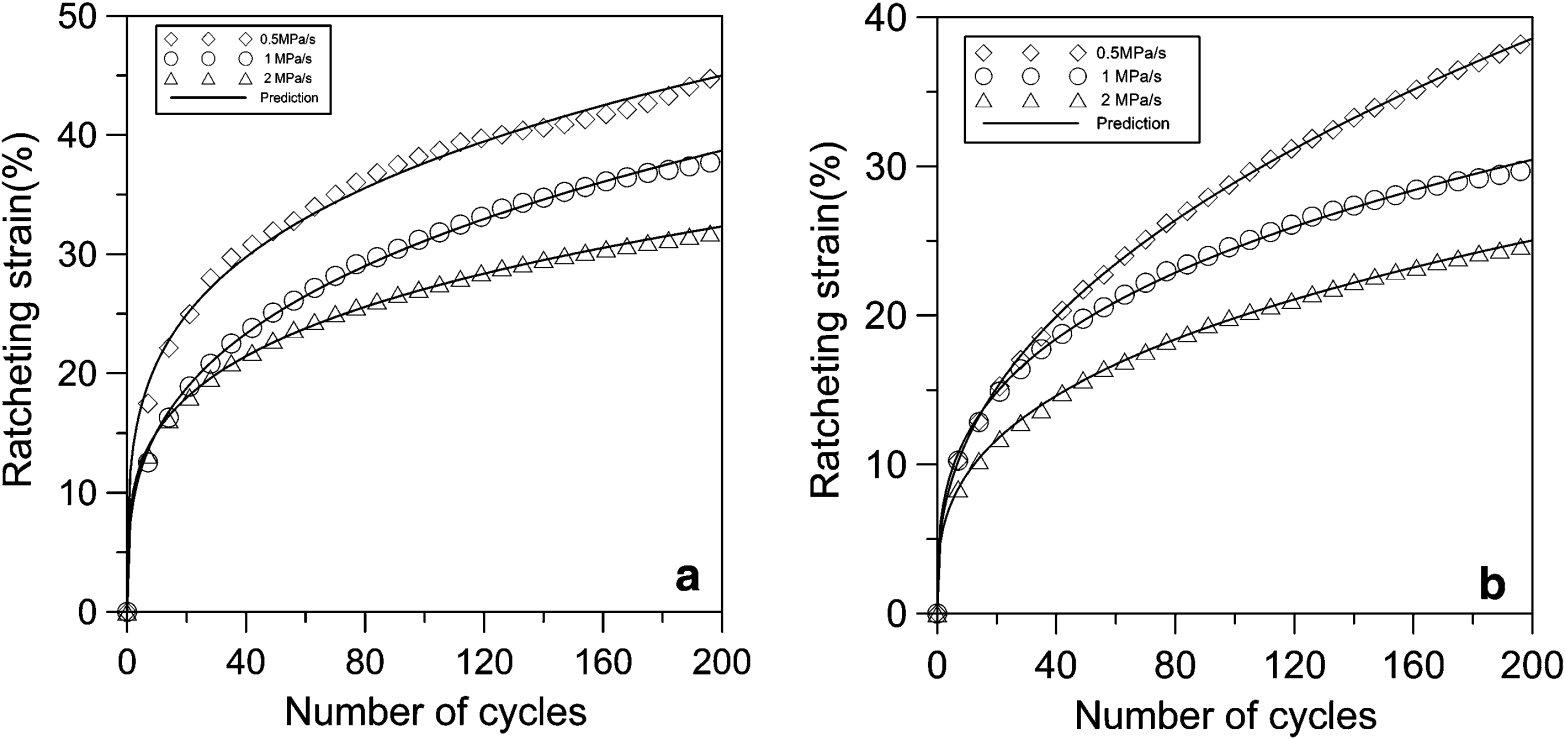
The ratcheting strain of cartilage at different stress rates. **a** Young cartilage; **b** adult cartilage

The ratcheting strains for young and adult cartilages with different stress rates were investigated by the experiments and the predictions of theoretical model. Figure 6 shows that the ratcheting strain curves of young and adult groups with different stress rates were inconsistent, which indicates that the ratcheting behaviors of young and adult cartilages are stress rate dependent. The ratcheting strains of articular cartilage for the two groups decrease with the increase of stress rate because the moving rate of collagen fiber within the samples may be smaller than the rate of employing force during a certain interval. It is noted that the ratcheting strain of young cartilage with different stress rates surpasses that of adult cartilage. When the cycle number is 200, the ratcheting strain of young cartilage with stress rate of 0.5, 1 and 2 MPa/s is 30%, 36% and 44%, respectively. However, the ratcheting strain of adult cartilage is 24%, 28% and 38% under the same condition. In the first 40 cycles, the accumulation rate of young cartilage’s ratcheting strain is significantly faster than that of adult cartilage.

The ratcheting strains of young and adult cartilages with different stress rates were predicted by the ratcheting model constructed in this study. It is found that the predictions match the experimental data very well for both young and adult cartilages as shown in Fig. 6.

### Effect of stress amplitude on ratcheting strain of young and adult articular cartilages

**Fig. 7:**
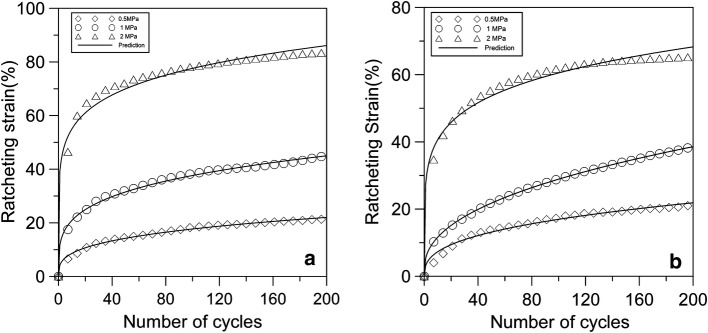
The ratcheting strain of cartilage with different stress amplitudes. **a** Young cartilage; **b** adult cartilage

The ratcheting strains for young and adult cartilages with different stress amplitudes were also investigated by experiments and predictions of theoretical model. It is noticeable for the effect of stress amplitude on the ratcheting strains of young and adult articular cartilages as shown in Fig. 7. Both the ratcheting strains of young and adult cartilages increase with the stress amplitude increasing. When the stress amplitude is 0.5 MPa, there exists a little difference in the ratcheting strain values between young and adult cartilage samples. The difference is becoming more apparent with increase of stress amplitude. When the stress amplitude is 2 MPa, the gap of ratcheting strain between them expands to 20% at 200 cycles as shown in Fig. 7. This result means that the young cartilage’s ratcheting deformation accumulation is greater than the adult cartilage’s with high stress amplitude.

The ratcheting strains of young and adult cartilages with different stress amplitudes were also predicted by the ratcheting model. It is found that the predictions agree with the experimental results very well for young and adult cartilages with different stress amplitudes.

### Ratcheting strain of different layers of young and adult cartilages

**Fig. 8:**
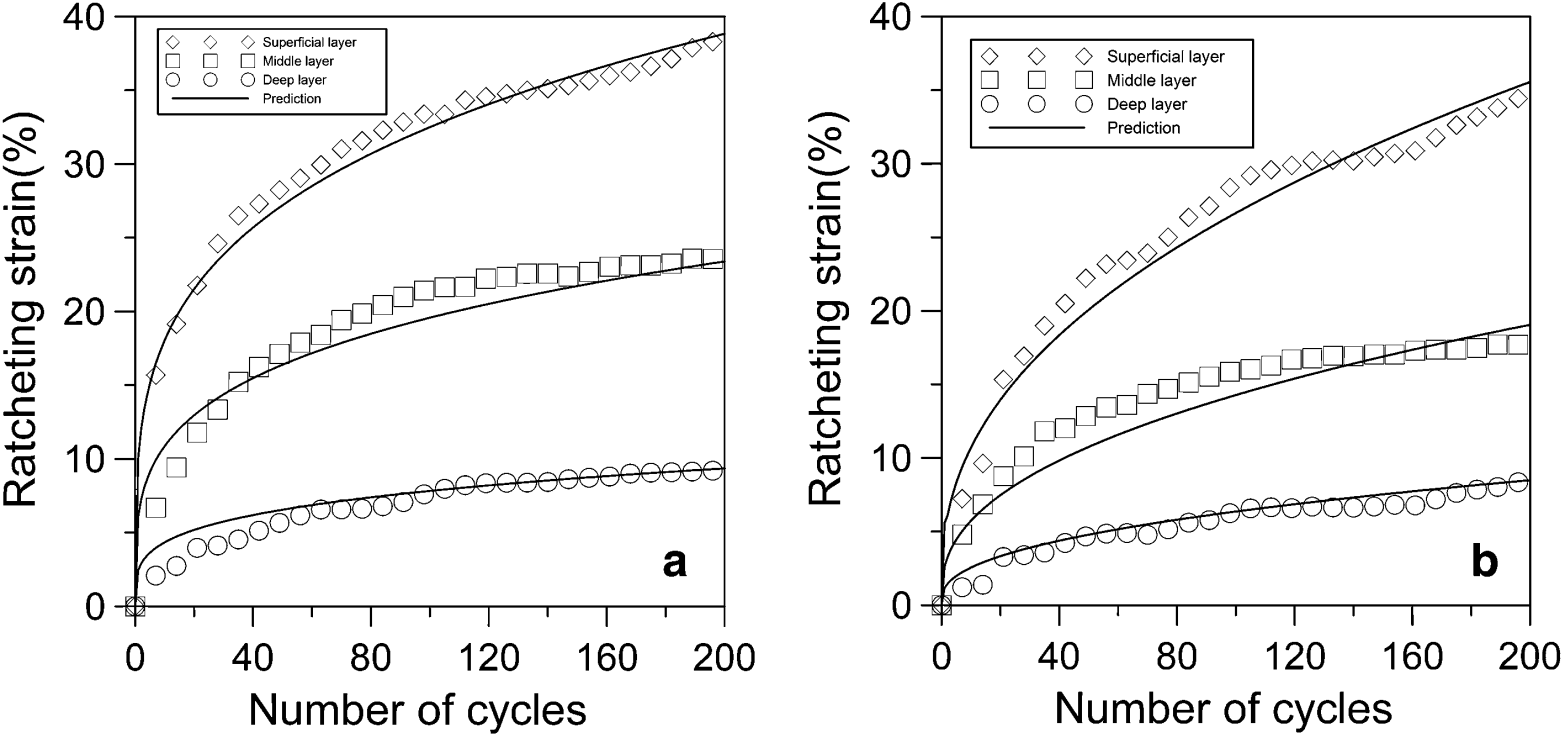
The ratcheting strains of different layers of cartilage with stress amplitude of 1 MPa and stress rate of 0.5 MPa/s. **a** Young cartilage; **b** adult cartilage

Considering the depth-dependent mechanical properties of articular cartilage, the ratcheting strains of different layers of young and adult cartilages were investigated by experiments and theoretical predictions, too. Figure 8 shows the ratcheting strains of different layers of young and adult cartilages with stress amplitude of 1 MPa and stress rate of 0.5 MPa/s. It is found that both the ratcheting strains of young and adult cartilages decrease gradually along cartilage depth from superficial layer to deep layer, which means that the ratcheting strain of superficial layer is the largest and the ratcheting strain of deep layer is the smallest, as shown in Fig. 8a, b. On the whole, the ratcheting strains of different layers increase with the ongoing compressive cycle and the gap among the three layers is widening. The rising rate of ratcheting strain in the superficial layer is the largest, and there is a slow increase of ratcheting strain in the deep layer.

**Fig. 9:**
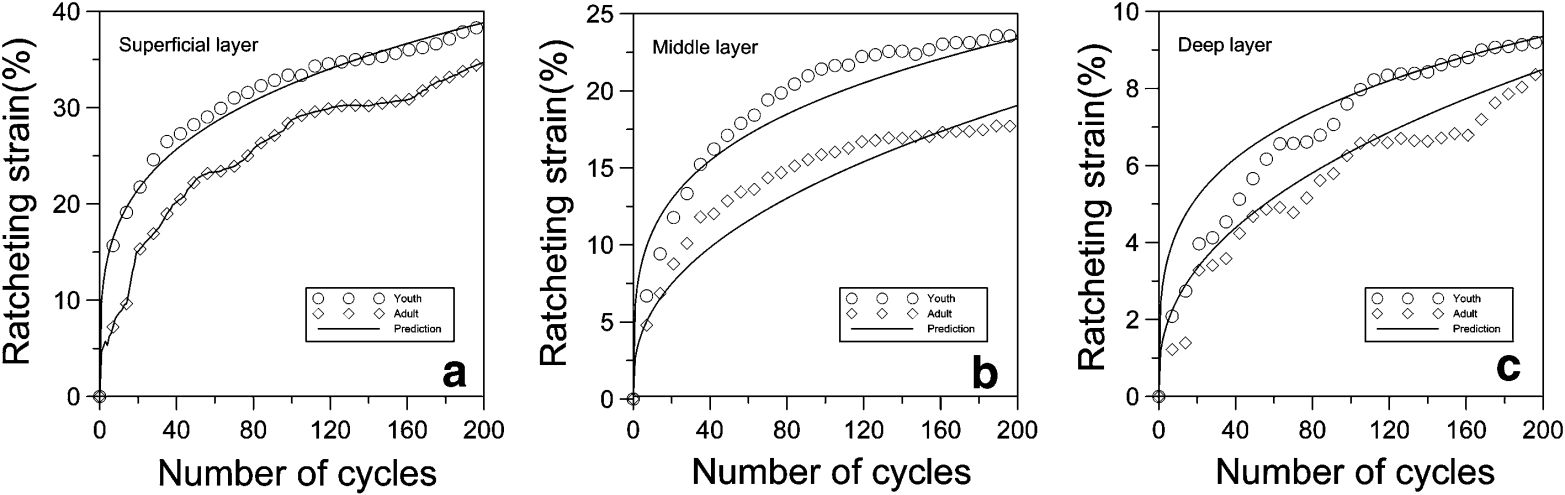
The comparisons of ratcheting strain of young and adult cartilages in the different layers. **a** Superficial layer of cartilage; **b** middle layer of cartilage; **c** deep layer of cartilage

Figure 9a–c shows the comparisons of ratcheting strain of young and adult cartilages in different layers. It is noted that the ratcheting strains of young cartilage in the three layers are greater than those of adult cartilage. When the number of cycle is 200, the gap of ratcheting strain between young and adult cartilages is 6% in the superficial layer, is 4% in the middle layer, and is 1% in the deep layer. The results show that the gap of accumulation rate of ratcheting strain between young and adult cartilages also decreases along cartilage depth from superficial layer to deep layer.

The ratcheting strains of different layers of young and adult cartilages were also predicted by the ratcheting model. Figures 8 and 9 show that the predictions of ratcheting strain agree with the experimental data very well, too.

## Discussions

Articular cartilage is the connective tissue covering the surface of subchondral bone in diarthrodial joints, playing a crucial role in transmitting loads, absorbing shock and sustaining daily loading histories. It is subjected to both static and dynamic loads up to ten times the body weight in the daily life [16, 17]. The study on mechanical properties of articular cartilage is of great significance to the prevention and treatment of cartilage disease and the development of artificial cartilage. The accumulation of ratcheting strain caused by cyclic compression can lead to cartilage fatigue, and further cause arthritis. This study investigated the ratcheting behaviors of young and adult cartilages with different loading conditions, and concluded that the ratcheting behaviors were different in different maturation levels of cartilage.

The ratcheting behaviors of both young and adult articular cartilages appear as two stages during the cyclic compression. The first stage is very short, and the ratcheting strain increases rapidly. The second stage is relatively long and the ratcheting strain grows slowly, showing a smaller strain accumulation rate as shown in Fig. 5. The ratcheting strain evolution of cartilage agrees with that of viscoelastic polymer materials [14, 18–20]. Figure 4a–d shows that the stress–strain response curves of young and adult cartilages are from sparseness to denseness. Under the same number of cycles, the stress–strain curve of young cartilage is sparser than that of adult cartilage. With the number of cycles increasing, this phenomenon is not obvious. Gofman et al. also found similar conclusions for the study of the mechanical properties of the swollen hydrogel composition “cellulose–polyacrylamide” [21]. And they found that the loading curve of the second cycle practically coincided with the unloading curve of the first cycle. This phenomenon is also found for young and adult cartilages and is shown in Fig. 4e, f.

The stress amplitude and stress rate generally have influence on the ratcheting behavior of material, which are the typical factors for articular cartilage during daily activities, such as walking and running. In 2015, we studied the ratcheting behaviors of adult articular cartilage and found that the ratcheting strain increased with increase of stress amplitude, whereas it decreased with increase of stress rate [7]. In this study, the same conclusions for adult cartilage are obtained, and the ratcheting behaviors of young cartilage were also investigated with different stress amplitudes and stress rates. It is found that the changing trends of ratcheting strain for young cartilage with different stress amplitudes and stress rates are the same as the results of adult cartilage. However, the ratcheting strain of young cartilage is larger than that of adult cartilage. It is noted that the ratcheting strain of young cartilage is a little more than that of adult cartilage with small stress amplitude. With increase of stress amplitude, the difference of ratcheting strain between young and adult cartilages is growing. Previous studies have shown that measured mechanical properties may also be affected by developmental variation in the structural features of the collagen network as well as aggrecan monomers and aggregates [22, 23]. Williamson et al. found that with growth from the fetus to the adult, the equilibrium and dynamic tensile moduli, and strength of cartilage samples increased by an average of 391–1060%, the collagen concentration (per wet weight) increased by 98% [12]. Gannon’s research results show that articular cartilage was found to become stiffer and less permeable with age, whilst collagen content significantly increased [13]. Therefore, the ability of adult cartilage resistance to deformation is stronger than that of young cartilage.

The inhomogeneous composition and structure of articular cartilage determine its anisotropic mechanical properties. The anisotropic mechanical behaviors of adult cartilage have been extensively studied under compressive, sliding and rolling load [24–27]. However, there are fewer studies on the anisotropic mechanical behaviors of young cartilage. The present study investigated the depth-dependent ratcheting behaviors of young and adult cartilages. Figure 8 shows that the ratcheting strain of young cartilage decreases along its depth from surface to deep, which is the same as the depth-dependent ratcheting strain of adult cartilage. The equilibrium stiffness of articular cartilage increases with increasing distance from the articular surface and this is linked to increasing osmotic pressures with increasing distance from the surface [28–31]. The young cartilage has a greater fluid content than adult cartilage. And it is noted that the ratcheting strains of different layers of young cartilage are larger than them for the adult cartilage as shown in Fig. 9.

The ratcheting behavior of material can be predicted by ratcheting model. Cai et al. proposed a universal ratcheting model (URM) to describe the relationship between saturated ratcheting strain and ratcheting stress [32]. However, the model could not describe the rate-dependent ratcheting behavior of material. In 2008, Zhang et al. modified the URM model by considering the rate-dependent ratcheting characteristic, and used it to predict the ratcheting strain of polytetrafluoroethylene (PTFE) with different loading rates [14]. In this study, we obtained the modified URM model which not only considered the rate-dependent ratcheting characteristic, but also considered the depth dependence of ratcheting behavior of cartilage. By applying the modified URM model, the ratcheting strains of young and adult cartilages with different stress amplitudes and stress rates were predicted, and simultaneously, the ratcheting strains of different layers of young and adult cartilages were also predicted. Finally, all the predictions were compared with the experimental data and the results show that there are agreements between them.

## Conclusions

This study elucidated the ratcheting behaviors of young and adult articular cartilages with different loading conditions and their depth-dependent ratcheting strains. With the cyclic compression going on, the stress–strain curves of young and adult cartilages become denser, and the hysteresis loop area of the young cartilage is greater than that of the adult cartilage, which means that there is larger ratcheting strain for young cartilage in the same number of cycles. The loading conditions, such as stress rate and stress amplitude, can influence the ratcheting behaviors of young and adult cartilages. The ratcheting strains of young and adult cartilages increase with rise of stress amplitude, while they decrease with rise of stress rate. It is noted that the ratcheting strain of young cartilage with different stress rates surpasses obviously that of adult cartilage, and, however, there exists a little difference for the ratcheting strain values between young and adult cartilage samples with small stress amplitude. The difference appears apparent with increase of stress amplitude. The ratcheting strains for not only young cartilage but also adult cartilage are depth dependent. It is found that their ratcheting strain in the superficial layer is the largest and the ratcheting strain in the deep layer is the smallest. The ratcheting model built in this study can predict not only the ratcheting strains of young and adult cartilages with different loading conditions but also their depth-dependent ratcheting strains very well. These results are important for analysis of cartilage damage and development of cartilage repair.

## Data Availability

The datasets used and/or analyzed during the current study are available from the corresponding author on reasonable request.
